# Structural clues to articular calcified cartilage function: A descriptive review of this crucial interface tissue

**DOI:** 10.1111/joa.13728

**Published:** 2022-07-22

**Authors:** Lucinda A. E. Evans, Andrew A. Pitsillides

**Affiliations:** ^1^ Department of Comparative Biomedical Sciences Royal Veterinary College, University of London London UK

**Keywords:** articular calcified cartilage, calcified cartilage chondrocytes, calcified cartilage matrix, cement line, osteoarthritis, tidemark

## Abstract

Articular calcified cartilage (ACC) has been dismissed, by some, as a remnant of endochondral ossification without functional relevance to joint articulation or weight‐bearing. Recent research indicates that morphologic and metabolic ACC features may be important, reflecting knee joint osteoarthritis (OA) predisposition. ACC is less investigated than neighbouring joint tissues, with its component chondrocytes and mineralised matrix often being either ignored or integrated into analyses of hyaline articular cartilage and subchondral bone tissue respectively. Anatomical variation in ACC is recognised between species, individuals and age groups, but the selective pressures underlying this variation are unknown. Consequently, optimal ACC biomechanical features are also unknown as are any potential locomotory roles. This review collates descriptions of ACC anatomy and biology in health and disease, with a view to revealing its structure/function relationship and highlighting potential future research avenues. Mouse models of healthy and OA joint ageing have shown disparities in ACC load‐induced deformations at the knee joint. This raises the hypothesis that ACC response to locomotor forces over time may influence, or even underlie, the bony and hyaline cartilage symptoms characteristic of OA. To effectively investigate the ACC, greater resolution of joint imaging and merging of hierarchical scale data will be required. An appreciation of OA as a ‘whole joint disease’ is expanding, as is the possibility that the ACC may be a key player in healthy ageing and in the transition to OA joint pathology.

## Graphical abstract

Synchrotron phase‐contrast x‐ray image of a non‐stained, non‐fixed ACC/HAC junction, in which both mineralised and unmineralised tissues are clearly visible. “Half‐in, half‐out” chondrocytes are highlighted by white chevrons. Sample originates from a 40 week‐old male STR/Ort mouse, from the Royal Veterinary College. “Half‐in, half‐out” chondrocytes were also observed in healthy and younger mice (authors' own unpublished data).

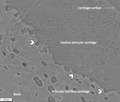

## 1 ARTICULAR CALCIFIED CARTILAGE—A STRUCTURAL OVERVIEW WITH POTENTIAL RELEVANCE TO OSTEOARTHRITIS

Articular calcified cartilage (ACC) is a layer deep to the hyaline articular cartilage (HAC) and superficial to the subchondral bone (SCB; Findlay & Kuliwaba, 2016). The hyaline cartilage of the immature epiphysis initially has two layers of proliferating chondrocytes, these being a deep layer which forms bone and a superficial layer which forms HAC. The ACC has been described as forming at sites where the equilibrium between calcium salt absorption and deposition is no longer balanced (Wang et al., 2009). ACC comprises hypertrophic chondrocytes inhabiting pericellular matrices within lacunae in a mineralised extracellular matrix (ECM; Loqman et al., 2010). Unlike either bone or HAC, healthy ACC is mostly considered to be non‐layered and structurally homogeneous, although its thickness varies across condyles (Wang et al., 2009) and mineralisation differences are described in horses (Doube et al., 2007). With 10–100 times the stiffness of HAC (Oegema Jr. et al., 1997), it is intermediate in stiffness between HAC and SCB (Mente & Lewis, 1994). Although less stiff than SCB and containing less inorganic hydroxyapatite (Mente & Lewis, 1994; Zhang et al., 2012), human ACC contains greater calcium salt concentrations than SCB (Wang et al., 2009; Zizak et al., 2003), though the degree to which this is true varies across species and, probably, individuals (Green Jr. et al., 1970).

The gradated layer properties between HAC and SCB are believed to facilitate layer adhesion (Wang et al., 2009), and yet the osteochondral junction itself is not an uninterrupted gradient of smoothly changing characteristics, as might be anticipated. Instead, the superficial ACC surface adheres to HAC at an undulating yet definite mineralising front interface, often referred to as the ‘tidemark’, although these terms are not wholly interchangeable (see Section 6). The deep ACC surface adheres to SCB at the osteochondral junction or ‘cement’ line which also undulates (Schultz et al., 2015; Wang et al., 2009). In this way, the ACC is tightly interlocked between its neighbouring bone and HAC layers, with two interfaces; an upper non‐mineralised:mineralised interface across a collagen type II continuum and a deep collagen type II:type I interface across the mineralised ACC and SCB phases. Disruptions of the clear, upper tidemark interface are a hallmark pathological feature of OA (Boyde, 2021). This review will describe ACC and its related structures in detail, containing a minimal degree of natural overlap due to the close associations between different ACC components.

**FIGURE 1:**
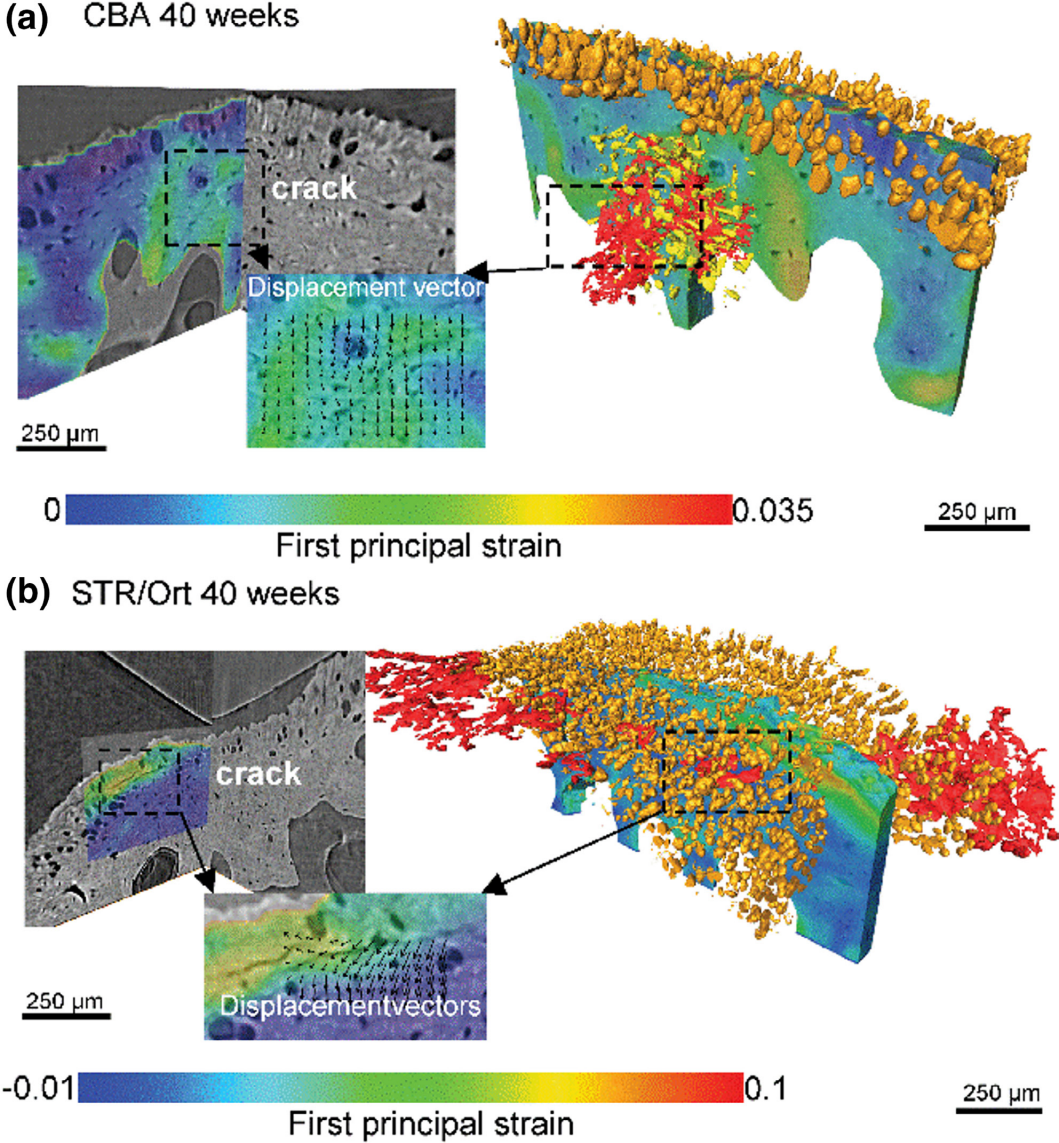
3D visualisation of ACC microcracking and its complex interaction with hypertrophic chondrocytes. (a) In the aged healthy control (CBA) mouse, cracks (red) develop in the subchondral bone where high tensile strains are found, interacting with the osteocyte lacunae (yellow). (b) In contrast, in an aged osteoarthritic (STR/Ort) joint, in association with highly localised tensile strains, the cracks were observed to intersect with the large pores left by the hypertrophic chondrocytes (orange) in the articular calcified cartilage. Reproduced with permission from Madi et al. (2019).

Some of the earliest anatomical changes currently identified in osteoarthritis (OA) are in the SCB (Bailey & Mansell, 1997; He et al., 2014; Kraus et al., 2018). This does not confirm bone as the ‘causal’ joint component, however, the mechanical environment and dynamics of a joint are determined by the anatomy of all constituent components, including the soft HAC in which there are also early (hydration‐related) changes (Maroudas et al., 1985). Transfer of locomotory forces through soft joint tissues and ACC into bone redirects and attenuates their energy. Therefore, anatomical and/or mechanical insufficiency of other joint tissues, including ACC, could potentially result in inappropriate force transference and in the bony symptoms (such as subchondral plate, SCP sclerosis and osteophytes) later observed. Evidence in support of this hypothesis has recently been reported by Madi et al. (2019) who have shown, through synchrotron imaging, substantial differences in the mechanical response of healthy versus OA‐predisposed murine ACC when subjected to mechanical loads (Figure 1). Although the STR/Ort mouse model used by Madi et al. (2019) is a spontaneous (rather than injury‐induced) model of age‐related progressive OA, similar in these respects to human primary OA, it cannot of course be assumed that findings in any inbred mouse model are directly informative of human biology or anatomy (Madi et al., 2019; Staines et al., 2017). Nonetheless, these data suggest that it is at least possible that mechanoresponsive changes in the ACC may precede those observed in the HAC or SCB—although whether this is true, and if true, whether such changes are causal or contributory to OA have not yet been confirmed—and the exploration of such changes may yet identify new and earlier imaging biomarkers of OA.

Literal molecular crosstalk between bone cells and chondrocytes in vivo is as yet unproven (Findlay & Kuliwaba, 2016), despite in vitro demonstrations of increased chondrocyte viability where they are co‐cultured (Amin et al., 2009). Nonetheless, solutes including humoral mediators can and do travel through the hard‐to‐soft tissues and vice versa (Arkill & Winlove, 2008). Additionally, communication between hard and soft joint components in the development of OA is evidenced by the fact that (in animal models) perturbation or protection of one compartment will often lead to corresponding changes in the behaviour of the other (Findlay & Kuliwaba, 2016). For example, OA induction by injection of vascular endothelial growth factor (VEGF) into HAC results in whole‐joint OA symptoms, including sclerotic bone where high VEGF levels are found (Hamilton et al., 2016). Likewise, surgically injuring bone or inducing bone‐specific increases in transforming growth factor‐beta expression will result in OA of all joint tissues including cartilage erosion (Baker‐LePain & Lane, 2012; Zhen et al., 2013).

## 2 LIMITATIONS TO RESEARCHING THE ACC

The ACC has been historically disregarded and is difficult to evaluate due to its slenderness. Nonetheless, all stresses passing through the HAC to cause local SCB changes must be conveyed through the ACC. Likewise, if OA is initiated in either of these tissues, their crosstalk must take place via ACC. As symptoms often arise in all condylar components (Loeser et al., 2012), it is indisputable that some such interactions occur. Attempts to examine ACC (or indeed, any joint component) using high‐resolution desktop micro‐computed tomography (μCT) are limited by restricted fields of view, low resolution relative to the very thin layer (particularly in mice) and excessive scan times. Lengthy scans are associated with movement artefacts, particularly in fresh or frozen tissues prone to relaxation, dehydration, thawing or decomposition over time. Tissue fixation can reduce sample motion but may introduce shrinkage artefacts and permanently affect material properties (Loqman et al., 2010). This means that such properties cannot be measured after scanning. Difficulty imaging intact ACC means that it is rarely assessed with consistent methodology in OA research. Some gross quantitative measurements of ACC, such as average thickness or tidemark roughness (TMR) across an entire condyle or joint have been undertaken, but not at resolutions required to discern microanatomical 3D changes (Findlay & Kuliwaba, 2016; Schultz et al., 2015; Wang et al., 2009). Horng et al. (2021) have described changes to HAC chondron orientation and shape in early OA human patellar and femoral subsamples by X‐ray phase‐contrast imaging, but what constitute ‘normal’ 3D microanatomical features over time have not been described in healthy ageing joints, specific animal models of OA or in the context of loading. This has meant that full evaluation of anatomical spatial ACC relationships to other bony, cartilaginous or miscellaneous changes remain enigmatic, as do any features that may have predictive qualities in determining future joint health.

Research into OA and more specifically cartilage is often rooted in the analysis of animal rather than human joints. Although highly valuable, animal models, including those of mice (Poulet & Pitsillides, 2010), rats (Bush et al., 2008), rabbits (Rytky et al., 2021), guinea pigs (Cai et al., 2018), dogs (Kiviranta, Tammi, et al., 1987) and thoroughbred horses unavoidably reduce the reliability of any conclusions drawn about human disease. Some nonetheless have benefits, particularly related to joint size—it is easy, for example to fit an entire mouse joint into a standard laboratory desktop μCT. Another practical reason for using animal models (rarely explicitly stated) includes the relative dearth of protective legislation. Likewise, as ‘pre‐OA’ cannot be screened for in human joints, it is impossible to confidently research this important state in humans, unless there is a massive financial commitment to longitudinal studies (spanning decades). Such studies would need to accept risks that technical changes (e.g. in scanning modalities) may render early data obsolete, as well as risking participant drop‐out, age and lifestyle behaviours that may compromise the objective comparability of OA/control groups. By comparison, any given inbred male STR/Ort mouse is essentially guaranteed to be pre‐OA before 16 weeks of age, and ‘late‐stage OA’ by 33 weeks, allowing research into the course of OA in a relatively compressed time frame, with fewer worries about the genetic heterogeneity and other complications of human research (Javaheri et al., 2018).

Selecting a suitable animal model for a given investigation is however complex because OA is a multifactorial disease with many potential aetiologies and endotypes, including aged, idiopathic and secondary OA (Deveza et al., 2019). An OA model that develops following surgical destabilisation of the medial meniscus, for example is likely to yield results more relevant to human secondary (injury‐induced) OA than to spontaneous age‐related OA (Haase et al., 2019). Likewise, animal models of age‐related OA are less likely to be relevant to injury‐induced OA. Animals also have very different lifespans and thus investigations into OA (or ACC) at a specific age in a rodent model will not necessarily allow any direct comparison to an equally specific human age—for example mouse growth plates (GPs) never fully fuse, unlike those of humans which close in adulthood (Staines et al., 2018). These differences require researchers to think laterally to identify the most appropriate time point at which human and animal data can be compared (if any).

Conclusions derived from animal‐based research cannot be blithely assumed to extrapolate to humans, and once an animal model is chosen, selecting a suitable control group is also vital.

Even when similarities between an animal model and human disease are later proven to be untrue, this does not mean that collected animal data are inherently irrelevant or unimportant. Animals do have both sentience and the legally recognised capacity to experience pain, so there are clear ethical arguments in favour of researching a cure for animal OA, even if the human situation is not directly improved by the pursuit (Birch et al., 2020).

Another limitation to assessing ACC and its relationship to HAC is that imaging modalities are generally sensitive to representing either soft or mineralised tissues, rarely both at the same time. Destructive histology is the easiest way in which to view both tissues simultaneously, but this results in the loss of 3D information and material integrity. Alternatively, stains are used to achieve contrast, which can introduce artefacts and often are used in association with low‐resolution CT scanning. The combination of histology and staining is useful in clearly defining the cement line and tidemark boundaries of the ACC, which can be otherwise unclear, but also damages the sample and yields only 2D information.

Although MRI is a common clinical imaging modality by which OA is assessed, it is unsuitable for assessing ACC. While visible with T1‐weighting, the ACC forms a bright band that exaggerates its thickness, likely due to the inclusion of the radial layer of HAC and a short T2 blurring effect, in which chondrocytes are not visible (Ma et al., 2019). Even a μCT study by Kauppinen et al. (2019) with a 2.8 μm effective voxel size relied on manual measurements and segmentation of histological slices in order to separate human ACC and SCB for individual analysis, presumably due to the close anatomical associations and similar mineralisation densities of each layer. Consequently, 3D detailed imaging of the ACC has been mostly retricted to high resolution, yet disruptive EM modalities applied only to small tissue segments.

There is a wealth of evidence to show that HAC is inhomogeneous through its depth, divisible into at least three specific zones (superficial, middle and deep) as a function of matrix structure and cellular characteristics (Mieloch et al., 2019). In contrast, there has been relatively little scrutiny to establish if this is also the case for ACC, although multiple tidemarks (when present) are recognisable evidence that ACC matrix mineral composition, at least, varies with depth (Doube et al., 2007). Nanoindentation studies are limited in their appraisal of ACC material properties, as they either indent with varying pressure through the HAC (Mieloch et al., 2019), or the HAC is removed which may affect the tidemark and thus biomechanics of ACC. Furthermore, sharp nanoindentation probes pick up moduli of specific macromolecules, which may become inhomogeneously distributed in OA, leading to unrepresentative results (Loparic et al., 2010; Mieloch et al., 2019).

The ACC and its components are frequently given a range of names, rather than being consistently described, which can create difficulty in searching the literature. For example, it may also be referred to as the calcified zone of cartilage (CZC; Schultz et al., 2015); zone of calcified cartilage (ZCC; Oegema Jr. et al., 1997); component of the chondro‐osseous junction (Lyons et al., 2005); the deepest layer of cartilage (potentially open to misinterpretation as meaning the radial, aka deep or basal, layer of HAC) or simply, calcified cartilage (CC), an umbrella term which, when used correctly, would encompass both articular and GP calcified cartilages. As scientific understanding of these regions and their respective involvements in pathology becomes more sophisticated, so must the use of language in delineating them suitably. The possibility remains that the under‐investigated ACC may serve crucial, hitherto undefined roles, in maintaining the integrity of both SCB and HAC.

## 3 MATRIX

The ECM of the ACC is synthesised by resident chondrocytes and comprises proteoglycans, minerals and type II collagen fibres. Mieloch et al. (2019) describe the overall total articular cartilage (TAC, HAC and ACC combined) as 70%–80% water, 15% collagen (mainly type II) and 9% aggrecan, a composition that varies with depth—the ACC is certainly less aqueous than the more superficial cartilage zones, with up to 75% of ACC dry weight being hydroxyapatite (Zizak et al., 2003). The remaining components comprise variable concentrations of calcium phosphate, other calcium salts (Zhang et al., 2012) and traces of minor proteoglycans and other non‐collagenous proteins. The ACC contains less type II collagen (measured by dry weight) than HAC and less hydroxyapatite than bone (Zhang et al., 2012). Collagen fibres in ACC are oriented parallel to the long bone axis, perpendicular to the HAC surface; many are continuous with the deep zone of HAC, likely facilitating layer adhesion across the tidemark. ECM of ACC is not typically considered to be structured in concentric lamellae (normally a bone‐specific feature), but Keenan et al. (2019) have described such lamellae in the ACC matrix of BALB/c wild‐type mice, visible in transmission electron and light microscopy.

### 3.1 Matrix thickness

ACC has been described as being approximately 20–250 μm thick (Hoemann et al., 2012), and a relatively constant ~3%–8% of total articular cartilage (TAC) depth (combined HAC and ACC), a figure derived from studies in mammals such as humans and dogs (Oegema Jr. et al., 1997). Horses provide a notable exception with relatively thicker ACC, in which 20% of TAC depth can be ACC in the equine metacarpal joint (Arkill & Winlove, 2008). However, close attention to published figures from Zheng et al. (2022) suggests that it may reach up to 60% of TAC thickness in certain regions of C57/BL6 mouse joints, a finding which could highlight either a specific outlier in this mouse strain, mice in general, or a significant effect of methodological differences on estimations of a thickness. Due to its location, the depth of ACC and the ratio of its volume to those of neighbouring tissues are thought to be maintained by a balance of bone remodelling and tidemark invasion (Oegema Jr. et al., 1997). The evolutionary history of human ACC is unclear and has not been researched in its own right, at least to the authors' knowledge—it is assumed to be a default consequence of the evolution of endochondral ossification as a growth mechanism.

There is intra‐specific variation in ACC depth, as is the case with HAC thickness and bone mineral density. ACC is substantially thinner than HAC in all imaged species, and within species, relative thicknesses of ACC to HAC as part of the SCP may be genetically determined. Loading experiences do appear to ‘fine tune’ individuals' ACC thickness to some extent, with Doube et al. (2007) finding that high‐intensity treadmill exercise correlated to increased ACC thickness in adult horses' middle carpal bones. The extent to which load‐related ACC thickness modulation occurs in humans, and the mechanisms underlying any such occurrence are unclear. Previous literature has described the ACC as approximating 5% of TAC thickness (i.e. HAC is 20 times thicker than ACC in physiological health; Oegema Jr. et al., 1997). However, this varies between bones and species (HAC can be 25 times thicker than ACC on the human femoral condyle; Hunziker et al., 2002), with equine ACC displaying clear regional differences (Doube et al., 2007; Martinelli et al., 2002), while HAC is only 4 times thicker than ACC in equine metacarpals (Arkill & Winlove, 2008) and canine ACC has relatively minor regional differences (Kiviranta, Tammi, et al., 1987), such that no firm conclusions have been drawn as to whether there is a functionally optimal depth relationship. Since the total thickness of condylar cartilage is generally proportional to mammalian body weight (Stockwell, 1971), the equine variation in relative ACC depth is intriguing in its indication that this layer does not necessarily scale in the same way.

That ACC is often conserved in its relative TAC thickness during growth may, however, indicate that it has an important biomechanical function in the transference of shear forces into bone. An example of the locomotory role of HAC thickness in the requirement for a given amount of cushioning per unit volume of bone in a given joint is supported by the finding that its depth increases in cattle with skeletal growth during adolescence while remaining constant in its relative thickness as the most superficial 30%–35% of the SCP (Taheri et al., 2019). Identification of a similar relationship for ACC may raise the question of whether atypical ACC thickness or SCP proportion is related to OA onset and/or progression. However, it has rarely (if ever) been investigated whether the thickness ratios of HAC to ACC are atypical in genetic models of spontaneous, age‐related OA, versus healthy controls. Indeed, in some studies, a greater relative thickness of HAC than ACC is implied to be a sign of physiological health by default (Cai et al., 2018), due to HAC thinning partly via tidemark invasion being an established component of OA. However, this indicates that thin ACC is in some way inherently healthy or protective against OA—a hypothesis which has not been confirmed, despite research suggesting that it may be a promising avenue of study. Rytky et al. (2021) have confirmed that ACC is thickest in the patella (subject to shear forces) and thinnest in the tibial plateau (subject to greater compressive loads) in New Zealand White rabbit knees. Omoumi et al. (2019) found that medial tibial plateau cartilage thickness differed more in human OA and non‐OA samples than elsewhere in the affected knee joints—this study did not or was unable to describe the ACC contribution to this finding, likely due to its poor visibility. The thickness of the ACC itself would change the layer's modulus (Mente & Lewis, 1994), and so these findings support a biomechanical hypothesis of ACC thickness determination which may yet prove to translate across species.

Tidemark and cement line undulations mean that ACC depth varies across any given joint region and is confirmed to vary widely across the tibial plateau (Schultz et al., 2015). Measurements are typically taken at a range of locations to obtain a mean thickness per sample, a technique which is standard in both histological and non‐invasive imaging studies (Kestilä et al., 2018; Staines et al., 2016). ACC thickness variance is often unreported, and so published results may not represent the full variety of thicknesses across the joint plateau. Likewise, ACC thickness measurements from osteochondral cores or isolated histological regions are therefore representative of a small area in an already small joint sub‐sample (Shorter et al., 2019). Recent studies assessing mean thickness values across the tibial plateau have found it to be thinnest in the peripheral region of the medial plateau and thickest at the medial edge of the lateral plateau, in both OA and healthy knees (Schultz et al., 2015). As human and mouse OA is most frequently located on the medial plateau, this raises the question as to whether local ACC thickness influences this predisposition—or indeed, if ACC thickness is modulated in a protective response to this region experiencing increased loading.

The average thickness of the ACC around specific condylar anatomical landmarks must be of relevance to regions which experience particularly intense shear forces during locomotion and other activities, yet has not been fully investigated. For instance, whether ACC is relatively thicker or thinner in regions above GP bridges—bony fusions connecting the epiphysis and metaphysis—has not been investigated to the authors' knowledge. This is an intriguing question as GP fusion will likely alter the joint surface biomechanics, and there is evidence that excessive bridging can precede OA in uninjured mouse models (Staines et al., 2016).

#### 3.1.1 Matrix bioactivity

ACC depth, although not necessarily its stiffness nor mineralisation, has been shown to increase in exercised beagles (Ferguson et al., 2008; Oegema Jr. et al., 1997) and horse tarsi (Tranquille et al., 2009), while its cellularity and alkaline phosphatase (ALP) activity decrease with disuse (Nomura et al., 2017). Thinner femoral ACC was also observed in immobilised canine knees (vs controls) by Kiviranta, Jurvelin, et al. (1987). Deng et al. (2016) have confirmed thicker ACC in the lateral tibial plateau of overweight adult humans to contain more chondrocytes than thinner ACC, but whether these are sufficient to explain the total volume increase seen with exercise, or whether volume increases simply maintain the number of chondrocytes per unit volume of ECM, has not been described. These findings demonstrate that, far from being an inactive embryological remnant, the ACC is metabolically active and responsive to environmental perturbation. Increases in ACC thickness are typically attributed to tidemark encroachment or multiplication into the HAC, and rarely—or never—reworking of the deeper osteochondral junction, which could theoretically generate multiple cement lines. Instead, osteochondral junction remodelling results in gradual thinning of the ACC with age, as osteoclasts (known as chondroclasts in cartilage) erode ACC adjacent to SCB, and osteoblasts replace it with bone (Boyde, 2021). Further work by Doube et al. (2007) in horses has identified the rate of chondroclastic resorption, as opposed to tidemark linear accretion rate, as the major determinant of ACC thickness in young horses. Simply put, chondroclasts appear able to resorb ACC faster than the mineralising front is able to advance. Tidemark reproduction/multiplication is relatively obvious, due to the clear visibility of multiple tidemarks in histology, which may be enhanced using tetracycline or calcein labelling. By contrast, there is no method to directly measure the chondroclastic resorption rate in situ (Doube et al., 2007).

Certainly, ACC behaviour and/or biomechanics may be involved in or even responsible for the OA observed in genetic animal models and in humans. The standard orientation of ACC collagen fibres has been confirmed in various imaging modalities, including but not limited to small‐angle X‐ray scattering (SAXS; Moger et al., 2007), electron microscopy (Kaab et al., 1998) and differential interference contrast microscopy (Broom & Flachsmann, 2003). This uniformly longitudinal orientation of ACC collagen fibres is one of the earliest things to change at equine OA onset: using SAXS synchrotron CT, Moger et al. (2007) confirmed that ACC fibres in OA equine metacarpophalangeal joints are less perpendicular than those of healthy controls, and more twisted towards regions experiencing the highest loads, even in areas without overt cartilage lesions. Presumably, this reflects a long‐term and age‐related response to loading, as fibril movements must almost certainly occur prior to collagen fibres from the overlying HAC becoming encased in the solid ACC matrix. Whether these observations also describe early OA changes in other animal models and humans has not been confirmed, likely due to imaging restrictions and the relatively more shallow ACC layer in other species.

Only recently have Arkill and Winlove (2008) found evidence of an ACC chemical composition gradient, indicating that it is not as uniformly homogeneous as generally considered. Using predominantly backscattered SEM, Boyde (2021) also reports that the ACC is more highly mineralised in the immediate vicinity of chondrocyte lacunae. Rytky et al. (2021) have noted that the ACC appears thinner in μCT slices than in registered histology slices, for which there are two immediately plausible explanations. Either ACC swells during processing for histology, and/or the deeper, ‘older’ ACC layers in their rabbit model are relatively more mineralised than shallower layers, and therefore less differentiable from bone in Hounsfield units. Until methods are established that allow measurement of factors such as hypertrophic chondrocyte orientation and/or 3D clustering at varying depths or anatomical regions of the tissue, many questions regarding ACC homogeneity will remain unanswered. Synchrotron X‐ray phase‐contrast imaging appears to be one of the most promising avenues by which these questions can begin to be addressed, with Horng et al. (2021) having recently published details of HAC chondrocyte shape, size and distribution from cylindrical cores of human joints.

Part of the reason that an up‐to‐date thorough characterisation of ACC would be incomplete is the limitations imposed by the use of 2D histology to evaluate layer features. Parameters that have been measured primarily using only a 2D histological approach at specific locations include TMR, matrix thickness and hypertrophic chondrocyte size and clustering (Taheri et al., 2019; Wang et al., 2009). These are often prioritised features as they remain reasonably quantifiable following sample decalcification and sectioning. ACC thickness and TMR findings show less agreement between multiple studies and animal models, perhaps due to differences in resolving power and methods used. For example, Deng et al. (2016) found ACC to become initially slimmer in early stage OA (likely attributable to raised chondroclastic resorption and/or SCB sclerosis), but thenceforward thicken continually during progression, whereas Evans et al. (1994) consider that the entire SCP may initially thicken in OA, with the later‐stage HAC changes a consequence of this original increased mineral deposition. Boyde demonstrates with SEM on the human OA femur that in some pathological regions, where ACC has eburnated entirely with the HAC, the bone surface is sometimes instead covered with replacement secondary fibrocartilage in human samples (Bailey & Mansell, 1997; Boyde, 2021). Fibrocartilage in OA is mainly type I with some type II collagen and is relatively unstable as a repair tissue (Hoemann et al., 2012). It is feasible that lower‐resolution imaging may mislead researchers to believe that this replacement layer is indicative of thickened cartilage, rather than cartilage which has been entirely destroyed. Such ambiguities highlight the need to improve our anatomical evaluation of ACC architecture in both healthy and OA joints.

#### 3.1.2 Matrix response to loading

Some research infers that there are textural differences in OA versus healthy ACC. A study of cadaveric human samples by Zarka et al. (2019) described different frequencies of ACC microfracture: results showed that OA cadaveric knees contained more cracking in the SCB than healthier knees, and that the reverse was instead true for cracks in the ACC layer. This supports a biomechanical role for ACC in force transference through the knee joint and begins to answer the highly interesting question of whether there is an ‘optimum’ depth within the SCP for such microfractures to occur—indeed, whether there could be a quantifiable difference between beneficial and harmful cracks in mineralised tissues. There are nonetheless alternative views concerning the incidence of naturally occurring microfractures in these locations. Views that suppose that microfractures are indeed a component of healthy bone homeostasis extend to also suppose that larger cracks pose a fracture risk. The extent to which this relationship—or its inverse—could exist in ACC is unknown. Due to the longitudinal collagen orientation in the ACC matrix, cracks that do form often (though by no means only) do so vertically. As the deeper HAC layers also have longitudinally oriented collagen fibres (perpendicular to the joint surface), vertical ACC cracks are able to propagate into the HAC as a tear. Cracks in the bone cannot readily do the same, as such cracks are less likely to be vertical, and those which do form are attenuated at the cement line change in collagen fibre orientation. Thompson Jr. et al. (1991) followed ACC fractures in dogs and noted that cracks, when formed, did not appear to either heal or progress in 1 year, but cell invasion did occur if they contacted the marrow cavity. Although it is worth noting that the loads by which these cracks were induced, were sufficient to result in an OA phenotype, this still suggests evidence both for and against a physiological role of ACC microfracture.

**FIGURE 2:**
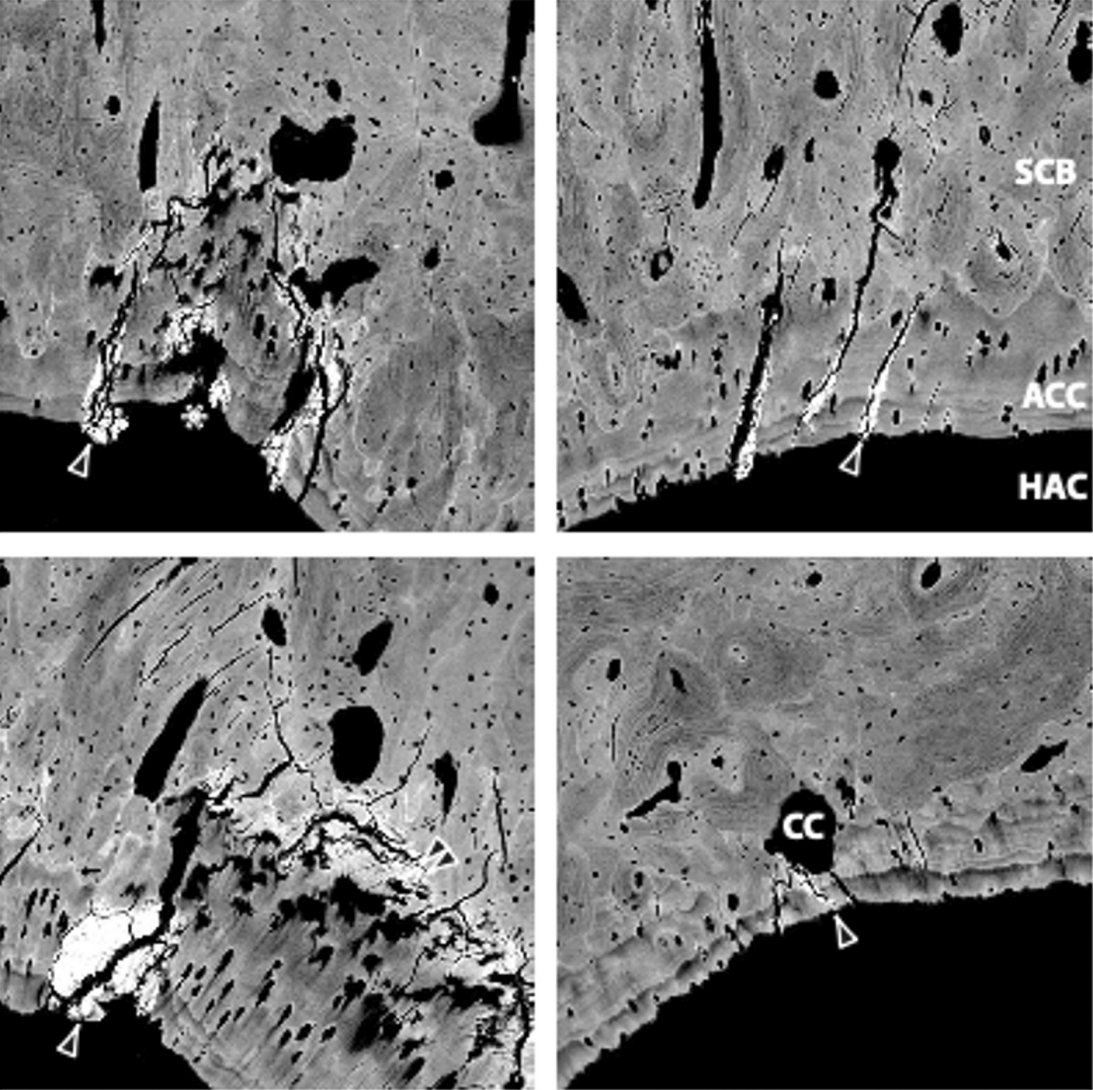
Amorphous mineralised material fills cracks in ACC and penetrates the mineralising front (arrowheads). Double arrowheads indicate hypermineralisation of osteochondral junction. Chondroclastic resorption (CC) removes crack filler materials. The mineralisation front may be damaged and delayed (*). ACC, articular calcified cartilage; HAC, hyaline articular cartilage; SCB, subchondral bone; field width 891 μm. Figure and legend reproduced with kind permission from Doube (2008).

In human SCP (ACC and SCB combined) as a whole, microcracks are found beneath eroded OA cartilage more so than in adjacent healthy regions, and these areas contain higher volumes of osteoid (Shimamura et al., 2019). This evidences the role of high‐impact load‐bearing in determining the regions most affected by OA in a given joint—loads result in both soft tissue erosion and hard tissue cracking. Osteoid is crucial to the repair process in SCB, but the repair process, or possible lack thereof, in ACC is relatively poorly documented, beyond the deposition of a high‐density mineral infill (HDMI) material to seal the defect (Laverty et al., 2015) (Figure 2). Horizontal cracks, uncommon in the ACC of healthy joints, as discussed (see Section 3.1.2), were found in the ACC of joints with severe OA and were a hallmark of especially fibrillated local HAC (Meachim & Bentley, 1978). Observations that describe SCP cracks as more common in OA have led authors to hypothesise that attempted crack repair by endochondral ossification is an initiating factor in local symptoms of OA, particularly in tidemark multiplication (see Section 6) (Sokoloff, 1993).

#### 3.1.3 Matrix composition changes in OA

The composition of the ECM in the ACC has been shown to change as OA progresses. This is due to both altered cellular metabolism, such that the matrix is less consistently maintained (and even actively catabolised by matrix metalloproteinases, MMPs; Hall, 2019), and direct downstream effects of whatever original insult initiated OA (e.g. fibrotic scar tissue formation after HAC‐penetrative injury or excessive loads; Sadeghi et al., 2015). These changes in composition can be presupposed to have textural consequences for the tissue (Deng et al., 2016). More chondrocytes with excessive levels of hypertrophy are likely in themselves to result in a relatively softened layer and have different phenotypic markers than their less‐hypertrophic healthy ACC counterparts. They secrete a matrix with increased collagen type X similar to the hypertrophic chondrocytes of the GP and also upregulate ACC Sclerostin (SOST) expression. This indicates that they play an arthroprotective role, as SOST knockout mice experience more severe OA for which their increased bone density does not appear solely responsible (Bouaziz et al., 2015). Despite the attempts of chondrocytes to maintain their ECM in OA, ultimately, the joint is compromised as the condition progresses.

## 4 CHONDROCYTES AND CHONDRONS

Chondrocytes in the HAC have been significantly more investigated than those in the ACC. As these chondrocytes are closely related, this section that mostly concerns the former supposes that changes in the behaviour of either are likely to shed light on their respective function in health and disease.

### 4.1 Development of mature chondrocytes

Briefly, chondroblasts initially form from mesodermal condensation and proliferate while secreting their own ECM and maturing into embryonic skeleton anlage. Joint shapes are formed during cavitation, resulting in an expanding cartilage ‘scaffold’, the majority of which mineralises through endochondral ossification to provide the template upon which bone is formed. Initially, the GPs remain cartilaginous, as do the ends of long bones. The calcified cartilage exists as a transitional layer between HAC and the mineralisation front, adjacent to the joint surface cartilage, as well as in layers abutting both ends of the GP. Later, with human skeletal maturity, full ossification of the GP means that only the articular layer of calcified cartilage remains.

Half‐lives for different molecular components of cartilage vary, but in general, type II collagen turnover is a slow process occurring over many decades, or even not at all, in healthy adult humans (Heinemeier et al., 2016). Proteoglycan turnover is much more rapid, with different components having half‐lives approximating anything from 30 days (Homandberg et al., 1996) to 25 years (Maroudas et al., 1998), yet the collagenous component is thought to be the principle target of irreversible change in OA (Tiku & Madhan, 2016). Data from juvenile mice suggest that cellular renewal arises through the continual, yet gradual proliferation of progenitor cells that are located in the most superficial layer of the HAC—the cartilage region most distant from the ACC (Li et al., 2017). Detailed cross‐sectional (rather than longitudinal) synchrotron images of human joints show how initially flattened, new chondrocytes gradually travel distally from this surface towards the ACC with age, with cells being more rounded and clustered in the intermediate zone, and are arranged in columns in the basal HAC layer (Horng et al., 2021). Chondrocytes with increasing levels of hypertrophy are found closer to the ACC layer, and during growth, become embedded in mineralised lacunae, creating the ACC itself, although the exact mechanisms by which the ACC (and the tidemark differentiating it from the HAC above) are formed remain unclear (Hoemann et al., 2012), despite this phenotype appearing in most if not all vertebrates with ACC existing between bone and HAC. Once embedded, the size of the lacuna itself (and pericellular matrix, PCM density) will constrain further size increase, unless there is the capacity for highly localised ECM remodelling. There is good evidence (at least in mice and rabbits) that hypertrophic chondrocytes of the GP chondroptose^1^ at the end of their life and do not travel into the bone to transdifferentiate into osteoblasts (Li et al., 2017; Pazzaglia et al., 2020). There is evidence both in support of and against these two potential outcomes for ACC chondrocytes at the cement line (Li et al., 2017). The process of mineralisation and tidemark advancement continues in the deep articular layers, but at a far slower pace than was previously occurring in the GP. Their close relationships mean that HAC chondrocytes are in many ways very similar to their hypertrophic ACC counterparts. In light microscopy, it is matrix composition, rather than cell differences, which make the division between these two zones most apparent. Whether new evidence for hypertrophic chondrocyte transdifferentiation to osteoblasts in the GP (Lui et al., 2019; Zhou et al., 2014) impacts this traditional view of the HAC:ACC chondrocyte relationship, as it has an appreciation of cell kinetics of endochondral bone growth, remains to be seen. The precise nature of the relationship between HAC and ACC chondrocytes remains poorly understood. 3D co‐cultures by Jiang et al. (2008) support the hypothesis that HAC chondrocytes inhibit their own mineralisation, suppressing ALP activity superficial to the tidemark. This behaviour is somewhat akin to that evidenced for osteocytes that are also believed to down‐regulate local mineralisation; in regions of excessive osteocyte death, the bone matrix becomes hyper‐mineralised, and thus brittle and fracture‐prone (Boyde, 2021). As ACC chondrocytes share many important features with both of these cell types, they may also have a role in preventing the over‐mineralisation of their local matrix tissue. Certainly, this would be consistent with observations both of chondrocalcinosis and hypocellularity occurring in OA (Fuerst et al., 2009; Mobasheri, 2002).

#### 4.1.1 Chondrocyte phenotypes

Chondrocytes have been reported to comprise approximately 3% of ACC by volume (Mieloch et al., 2019). Thicker ACC layers contain numerically more chondrocytes (Deng et al., 2016), but the lack of 3D data means that it is unclear whether the matrix:chondrocyte volume ratio is maintained as ACC depth increases. Chondrocytes anabolise and catabolise the HAC matrix (Hall, 2019). In OA, there is evidence of chondrocytic dedifferentiation resulting in a fibroblastic or hypertrophic phenotype, less capable of making a structurally sound matrix (Hall, 2019; Poulet et al., 2012; Staines et al., 2013). The development of these phenotypes is associated with ageing, accumulation of senescent chondrocytes, and reduced responsiveness to growth factors. Such cells have altered patterns of protein in their corresponding secretome, which has major implications for the cartilage tissue as a whole since chondrocytes communicate via secreted molecules as well as the cell to cell contact (Carpintero‐Fernandez et al., 2018; Loeser, 2002; Sanchez et al., 2017).

As with ACC, the predominant proteoglycan in healthy HAC is aggrecan—however, unlike ACC, the type II collagen‐rich ECM of HAC is completely unmineralised, instead having a softer and more aqueous composition. Although opinions differ regarding whether it is a cause or consequence of OA, many studies into OA symptoms in the HAC compartment have indicated that chondrocyte dedifferentiation comprises a core component of OA pathology (Hall, 2019). Chondrocyte dedifferentiation is marked by the synthesis of alternative proteins and signalling molecules, inappropriate for mature cartilage but which were suitable when synthesised prior to skeletal maturity as part of the GP/endochondral ossification process, and the recapitulation of early developmental patterns of behaviour (Pitsillides & Beier, 2011). This phenotype can substantially compromise the molecular composition of the matrix, for example chondrocytes returning to a relatively GP‐like state of hypertrophy produce MMPs: during skeletal growth, these are essential molecules which allow endochondral ossification by degrading the obsolete cartilage scaffold. In an adult skeleton with OA, however, they ectopically degrade healthy cartilage components. In turn, this alters the joint's mechanical properties, exacerbating OA and potentially thinning the cartilage to the point of bone‐on‐bone‐related symptoms (primarily pain). In elderly patients, the dedifferentiated phenotype of many chondrocytes is further exacerbated by the presence of cells in the local area approaching senescence and secreting pro‐inflammatory mediators associated with ageing, such as interleukin 1‐beta. This stimulates the production of more MMPs (particularly, MMP13) from those cells which have begun hypertrophy (Toh et al., 2016).

#### 4.1.2 Collagen production and clustering

Collagen production is also modified in OA, with increased quantities of type I collagen—less suitable for cartilage than bone—resulting in weak repair of any ECM defects (Styczynska‐Soczka et al., 2021). Chondrocytes shifting to a more fibroblastic phenotype also reduce type II collagen production (Hall, 2019), which again is more characteristic of a ‘healthy’ chondrocyte transitioning to hypertrophy in the GP, than of one inhabiting mature HAC. The resulting decreased type II:I fibril ratio is more characteristic of a proliferative layer chondrocyte in the GP. It is perhaps unsurprising, then, that chondrocyte over‐proliferation is also associated with OA, resulting in ‘clusters’ of abnormal chondrocytes in the affected cartilage, further compounding the issues. Clustering is considered by some to be a consequence of growth factors entering via fibrillated HAC (Hall, 2019). This results in proliferation of cells, the progeny of which are clustered, rather than the movement towards one another of pre‐existing cells. This is evidenced by clusters being particularly localised to HAC fibrillation (Lotz et al., 2010). Clusters have been observed in non‐OA mouse cartilage, although relatively infrequently, and Li et al. (2017) propose that physiological clustering may be related to tissue reshaping during growth.

Consistent with a hypothesis of over‐proliferation in OA, the number of ACC chondrocytes has been shown to significantly correlate with OARSI grade (Deng et al., 2016). The cell density of the ACC across 2D sections has been reported as 51 cells/mm^2^, significantly fewer than were found in the HAC, 152 cells/mm^2^ (Wang et al., 2009). However, throughout the TAC, the number of chondrocytes appears to decrease as OA progresses, resulting in overall hypocellularity (Bobacz et al., 2004), though not all studies have found that cell density decreases with OARSI grade (Shimamura et al., 2019). The chondrocytes within the HAC appear to be equally dense in their distributions throughout the layers between OA and healthier groups (Bobacz et al., 2004).

The clustered chondrocytes often also have an increased volume, indicative of OA (Hall, 2019). Chondrons in deeper cartilage in particular are found to be larger and less spherical with OA (Kestilä et al., 2018). Unfortunately, in 3D image analyses, resolution and threshold‐based segmentation can be insufficient to examine individual cells. Thus, chondrocyte clustering could cause larger lacunae containing multiple cells to be resolved as single enlarged pores, because when chondrocytes cluster, the lacunae may coalesce to make a large lacuna. This would lead to the incorrect conclusion that individual chondrocytes were larger and less spherical—though it may not actually be the case. Watershed operations, an image transformation method based on treating images as topographical maps, are effective at separating two items (such as two lacunae), which are directly in contact and initially appear to be a single item—they are therefore one potential method by which to minimise this effect (Palka et al., 2016; Sun & Ren, 2005). However, watershed operations may fail to separate some very closely associated items (Boudina et al., 2022), and/or also potentially result in the ‘opposite’ artefact of single abnormally shaped chondrocytes being analysed as multiple relatively spherical cells, since algorithms may be over‐sensitive to local minima and require uniform tolerance inputs (Sun & Ren, 2005). The use, limitations and advantages of watershed operations on standard μCT data are well explained in Method Note 75 (MN075) for the SkyScan 1172 desktop μCT (Bruker, Belgium). Limitations to watershed operations apply to any imaging modality which uses the mineral phase alone (the lack thereof representing chondrocyte lacunae) to assess ACC chondrocyte morphology. With manual segmentation of all chondrocytes in the ACC of even one bone being a near‐unthinkably time‐consuming task for any researcher, machine‐learning algorithms are likely one of the most promising ways in which to surpass these limitations (Horng et al., 2021). Another method of assessing the shape and clustering of chondrocytes is confocal laser scanning microscopy, used by Karim et al. (2018) to confirm that human OA chondrocytes become more clustered than non‐OA controls, sharing their lacunae and pericellular matrices. The volume of individual chondrocytes also increases, associated with cytoskeletal reorganisation, as does the total volume of clusters formed (Hall, 2019; Karim et al., 2018). These observations are from HAC and seem most marked in the superficial layer. Kestilä et al. (2018) assessed the effects of OA on chondrons using μCT of dehydrated osteochondral cores harvested from the tibial plateau. Their research confirms that OA‐affected chondrons decrease in sphericity as OA progresses, but unfortunately, the ACC could not be included in this study (Kestilä et al., 2018). ACC chondron sphericity, therefore, has not been quantitatively evaluated in 3D and their behaviour in both health and disease remains mysterious. Nonetheless, many papers state that chondrocyte hypertrophy increases in OA without reference to the inclusion or exception of those in the ACC (Dreier, 2010).

In addition to proliferation and release of MMPs, dedifferentiating OA chondrocytes become hypertrophic and produce type X collagen and ALP (Pitsillides & Beier, 2011) which are both markers of hypertrophy in the GP and in the ACC. Together, the altered secretions and behaviours mentioned indicate that the cells are reverting to an increasingly endochondral phenotype. Note that hypertrophy markers, inappropriately expressed in mature HAC during OA, are expressed physiologically in healthy ACC. Therefore, it could also be considered that HAC chondrocytes, as opposed to dedifferentiating, are in some respects undergoing accelerated ageing, and becoming prematurely more like ACC chondrocytes. ALP, type X collagen and VEGF are expressed in this layer in healthy joints (Nomura et al., 2017). Chondrocytes may in this respect be associated with ectopic osteophytic bone growth where type X collagen and other endochondral markers are expressed (Oegema Jr. et al., 1997). Interestingly, SOST knockout mice continue to develop osteophytes (Bouaziz et al., 2015) in OA, confirming that chondro‐neogenesis is the process by which at least some of these structures form (Aigner & Stove, 2003). Further molecules are also implicated in driving chondrocyte behaviour in OA but are somewhat beyond the scope of this review (Hall, 2019; Pitsillides & Beier, 2011).

#### 4.1.3 Chondrocyte regulation

Increased chondrocyte volume can indicate excessive fluid uptake, a hypertrophic phenotype, a stage of chondroptosis, cytoskeletal changes or a combination of the previous (Hall, 2019). Understanding the normal anatomy and volume of these chondrocytes, as well as their usual cell volume to matrix ratio is vital for differentiating these pathologies and disentangling which are indicative of repair processes, successful or otherwise, and which of an OA initiation or transition to end‐stage OA. However, the lack of high‐resolution 3D imaging of ACC chondrons, combined with aspherical cell morphology, means that chondrocyte volumes are generally measured as a mean estimation based on 2D histology. A stereological approach to these measurements might yield more accurate results of such measurements, as has been the case for chondrocyte number and HAC volume (Noorafshan et al., 2016), but in terms of chondrocyte volume, standard deviations may account for a relatively large proportion of the data obtained, perhaps because of the multiple populations of chondrocytes (in terms of non‐Gaussian distribution of size) observed by Li et al. (2017) located in the deep zone of cartilage.

The ACC typically has a significantly greater osmolarity than HAC, in excess of 450 mOsm (Maroudas & Evans, 2009). In OA, the osmolarity of synovial fluid decreases (from ~301–404 to 280–297 mOsm) resulting in a hypo‐osmolar environment and an increase in cartilage hydration (Hall, 2019). In the GP, local osmolarity also partially determines chondrocyte swelling as a consequence of water uptake (Hall, 2019), but increases in intracellular osmolarity are a more significant consideration in cell swelling at the hypertrophic zone (Bush et al., 2010). In culture, hypo‐osmolar changes in the chondrocyte medium cause reduced protein synthesis and increased cell swelling (Urban et al., 1993). In HAC, the degree of cell swelling is thought to be modulated by the mechanical effect of type VI collagen in the PCM (Aigner & Stove, 2003), and this notion can conceptually (not yet tested experimentally) be extended to the PCM of chondrocytes in the ACC, these matrices also being rich in type VI collagen. The ability of cells to swell indicates that lacunar walls are not ordinarily distended to their maximum by their chondrocyte and PCM contents. It is possible that this swelling results in only a very localised increase in lacunar diameter and consequent local pressure increase in the surrounding ECM, without a more widespread volumetric change of the cartilage. However, it may instead or also be the case that the entire volume of cartilage tissue increases with the increases in cell volume (assuming that the superficial layer of cartilage is also not itself maximally distended). Maroudas et al. (1985) have clarified that cartilage swelling is a component of human OA and can be used as a marker of collagen damage, consistent with canine findings of increased proteoglycan synthesis and HAC hydration at the earliest stages of OA (Carney et al., 1984), but Bank et al. (2000) primarily attribute this swelling to degradation‐related stiffness decrease of the extracellular collagen network, which allows the imbibing of additional fluid by the existing proteoglycans—rather than the combined effect of many cells pathologically increasing in volume.

The solidity of mineralised matrix around ACC lacunae, however, does imply a stricter regulation of cell swelling, even if they may displace the fluid PCM, within limits that would at least be temporarily defined by lacunar edges, until or unless local remodelling and/or PCM degradation were to occur. Thus, the relationship between cell volume increase and local hydration indicates that excessive hypertrophy may also reflect local proteoglycan/PCM/ECM efficacy. Local pressures associated with cellular difficulty in swelling beyond the lacunar‐defined maximum size have been hypothesised to be a cause of the chondrocyte cytoplasmic processes observed to extend beyond the lacuna and into the ECM, observed by Bush and Hall (2003) and further examined using confocal laser scanning microscopy (Karim et al., 2018). In this research again, however, cell volume increases were shown to be most significant in superficial HAC layers, and it is unclear whether further swelling of the already‐hypertrophic chondrocytes in deeper ACC layers is in fact a hallmark of OA. Further research confirms that ACC chondrocyte hypertrophy is not purely due to increases in water content, but is in fact accompanied by structural cellular changes and growth (Bush et al., 2008), which in themselves foretell a change in secretory phenotype (Hall, 2019). With chondrocytes being the sole cell type responsible for synthesis and maintenance of their ECM, changes to their phenotype, including the release of hypertrophy‐associated MMP13 (Hall, 2019) and/or an increased risk of cell death induced by mechanical loading, can also impact the local matrix composition in the long term (Hall, 2019).

#### 4.1.4 Chondrocyte morphologies

Aside from the standard changes to shape and dimension that occur with progression through the HAC and GP, six main chondrocyte morphologies have been described: normal, with short processes, with long processes, swollen, and clustered (Hall, 2019; Karim et al., 2018). Whilst clusters and chondrocytes with long processes are associated with OARSI scores >0 (Hall, 2019; Karim et al., 2018), it remains unclear how the processes (not to be confused with the chondrocyte primary cilia (McGlashan et al., 2008)) develop. One hypothesis could be that localised load‐related damage or PCM weakness causes a negative extracellular pressure gradient, which ‘sucks’ the cell membrane and cytosol into projecting outwards; alternatively, the damage may simply create a space in the surrounding area into which processes can actively extend (Hall, 2019). Chondrocytes in OA tissue themselves may be stimulating the release of catabolic enzymes that break down the local PCM, inducing the existence of this space (Hall, 2019; Karim et al., 2018). Chi et al. (2004) have observed processes connecting ‘paired’ chondrocytes in the superficial zone of adult rabbit cartilage, which somewhat controversially suggested to allow intercellular communication and is consistent with the observation by Mayan et al. (2013) that adult human chondrocytes may communicate via connexin43 gap junctions, connexin43 also having been demonstrated to be elevated in chondrocytes derived from the superficial layer of osteoarthritic cartilage (Mayan et al., 2013). Determining the origin of chondrocyte processes is particularly difficult in ACC, due to the lack of methods allowing the chondrocyte/lacunar interface to be clearly imaged in 3D, but their importance may be as indicators of altered biomechanics, cell metabolism, PCM composition, or vulnerability to cell death.

In 3D cell culture in agarose, the number of processes changes in response to osmolarity or fetal calf serum concentrations which directly influence local stiffness, potentially supporting a biomechanical hypothesis for their development (Karim & Hall, 2016, 2017). In OA, they may represent an attempted repair mechanism: chondrocytes also develop processes after cartilage is subjected to a scalpel cut in the presence of fetal calf serum (Karim & Hall, 2016). Processes do not appear to significantly increase the total volume of an affected chondrocyte (Bush & Hall, 2003), but there may be other benefits to the increased surface area in terms of local molecular deliveries. Culturing chondrocytes in media with varying mechanical pressures can also modulate their dedifferentiation (Hall, 2019), supporting a role for the load as a major determinant of morphology, as do the localised lesions seen in specific loading protocols (Poulet & Pitsillides, 2010), and in individuals with OA secondary to regional overloading (Kulkarni et al., 1998). Chondrocytes themselves affect the overall Young's modulus of the ACC, and therefore their own biomechanical environment.

One parameter measured in rare 3D studies of HAC is ‘sphericity,’ (quantified deviation from a perfect sphere). Changes to the smoother spherical or elliptical chondrocyte surface (Hall, 2019) in healthy cartilage, relevant to their cartilage depth (Horng et al., 2021), indicate that they become more cylindrical (Kestilä et al., 2018) or alternatively develop short or long processes in OA (Hall, 2019). Determining which of these is more frequently true will ultimately require quantitative studies at higher resolution, measuring multiple chondrocyte shape parameters. Such research may identify whether sphericity or other shape markers are suitable surrogate measures of de‐differentiative phenotype development, which would negate the need for staining or analysis of cell secretions to identify regions in which chondrocytes are adopting inappropriate GP or ACC‐like behaviour, as discussed previously (see Section 4.1.1.). In this regard, it is notable that chondrocytes in HAC deep zones are found to be significantly less spherical and larger in human tibial plateau samples with higher OARSI grades (Kestilä et al., 2018). It would be interesting to see whether this relationship is also apparent in ACC, whilst bearing in mind that cell shape itself may be less important than the cytoskeletal structure and any subsequent consequences in terms of second messenger signalling. Existing literature does show that the relationship between cell shape and metabolism is not so straightforward that conclusions about each can be drawn directly from the other, however valuable it may be to investigate them both (Parreno et al., 2017; Styczynska‐Soczka et al., 2021).

Although the sphericity of HAC chondrons is lower in human OA (Kauppinen et al., 2019), overly spherical chondrocytes fail to thrive in synthetic osteochondral grafts (Cooke, 2018). Determining optimum chondrocyte sphericity is thus a real consideration in the synthesis of hydrogel‐based implants for OA (Cooke, 2018). Identifying the ‘ideal’ chondrocyte sphericity would also improve osteochondral allograft success rates, allowing the selection of the healthiest non‐OA osteochondral regions. Such innovation may even enhance procedure availability, with contraindications overcome by an improved selection process: currently, failure rates (~18%) are reduced by a focus on limiting recipients to those with certain characteristics associated with a lower risk of failure (Cavendish et al., 2019). However, donor tissues can more feasibly be visualised in high resolution than those of recipients.

Ultimately, whether OA is a consequence of chondrocyte morphology or a cause of its alteration is frequently speculated upon, but will remain unclear until OA aetiogenesis is more fully understood. Research into ‘the healthiest’ ACC anatomy in particular is required before implants can be designed for optimal function, instead of the current trial and error approach to OA treatments. Certainly, chondrocyte tolerance to directional stress is sensitively tuned (Healy et al., 2005), with low shear being actively chondroprotective (Carter et al., 2004)^,^ whereas high shear can result in apoptosis and matrix degradation (Lee et al., 2003; Trevino et al., 2017), raising the possibility that tidemark undulation plays a protective role in load transference. In this case, an easy and automated method for measuring TMR across an entire joint quickly and easily would be highly valuable for researchers with potential clinical applications in the future.

## 5 GP SIMILARITIES AND DIFFERENCES

Non‐ACC is also found in long bones at the GP, a transverse disc separating the epiphysis from metaphysis in all species with secondary centres of ossification (Xie et al., 2020). The GP is of interest in OA, as researchers have frequently suggested that OA is at least partly a consequence of HAC chondrocytes and tissues adopting an ectopic ‘GP phenotype’ (Pitsillides & Beier, 2011). For example, while GP chondrocytes normally produce type X collagen, this occurs during OA pathology in the HAC, as a consequence of cellular dedifferentiation. It may thus be beneficial to consider how these similarities between AC pathology in OA and the GP phenotype impact our understanding of the ACC.

During growth, GP chondrocytes proliferate and hypertrophy to facilitate longitudinal bone expansion. In humans, the GP begins to mineralise at skeletal maturity, such that the two bone regions ultimately become completely fused. In rodents, mineralisation is incomplete, but much of the GP cartilage is nonetheless gradually replaced by bony ‘bridges’ (aka ‘tethers,’ Chen et al., 2009) which similarly solidify the epi–metaphyseal connection. The epiphysis contains the secondary ossification centre, the GP having been sandwiched by its formation following primary ossification at the diaphysis. The secondary ossification centre originally forms from GP chondroprogenitor cells and is hypothesised to have evolved to mitigate the intensity of shear forces on the GP during bone growth and loading (Xie et al., 2020). Research has rarely (if ever) however addressed whether the biomechanical forces engendered at the articular surface or ACC, specifically, are changed as a direct consequence of GP fusion/bridging.

While the GP is spatially disconnected from the articular ACC, there are many aspects of its phenotype and development that have been characterised sufficiently that we understand the articular cartilage better for it. During ACC development, its resident chondrocytes behave in many ways akin to those of the GP (Oegema Jr. et al., 1997); they are, after all, both growth cartilages in a young growing mammal. In OA, the rapid advancement of the ACC mineralising front into HAC is behaviour akin to a GP (Oegema Jr. et al., 1997), even though the same process, occurring gradually throughout life and counterbalanced by equally gradual superficial chondrocyte proliferation and differentiation, may be a normal part of osteochondral junction maintenance. Likewise, chondrocyte hypertrophy is a necessary component of endochondral GP ossification, and normal phenotypic behaviour in ACC, but appears to become exaggerated and inappropriately located in late‐stage OA HAC (Hall, 2019).

The chondrocyte hypertrophy embarked upon at the ACC and within OA cartilage nonetheless has some differences from endochondral ossification and osmotically‐driven cell swelling. The volumetric dimensions of osmotically swollen cells are different to those of GP lengthening cells (Bush et al., 2008). Although this is to be expected given the differing constraint from the ECM of both regions, directionality and anatomy are important features to consider when making comparisons between chondrocyte volume in each circumstance. Estimations of chondrocyte volume based on 2D visualisation are more common than 3D analyses of cell shape and orientation. This is likely due to both the time‐consuming, manual analyses required and concerns that cell shape is artefactually altered during tissue processing, fixation and histology—as well as histology being 2D by nature. In the GP, chondrocytes are ellipsoid in the proliferative, and more spherical in the hypertrophic zones (Loqman et al., 2010). In OA, hypertrophy in the HAC is linked to reduced sphericity (Kestilä et al., 2018), and in this respect, OA‐related hypertrophy does not resemble GP chondrocyte hypertrophy.

In the GP, there are changes to membrane transporters that affect cellular ion uptake and thus volume, in the absence of local osmolar change. NKCC (Na–K–Cl cotransporter) stimulation can lead to increases in cell volume, resulting in hypertrophic zone chondrocytes (Bush et al., 2010), which later undergo ‘chondroptosis,’ involving extrusion of cytoplasmic components, as part of a controlled cell death which is also signified by a nuclear breakdown with high levels of autophagy and lysosomal activity (Hall, 2019; Pazzaglia et al., 2020). Similar chondroptotic changes in the articular cartilage are also observed in surgery‐induced OA models (Aigner et al., 2004; Hwang & Kim, 2015). Macrophages, rather than chondroclasts, are active at the GP in clearing the consequent debris (Pazzaglia et al., 2020).

We hypothesise that cartilaginous joint areas, perhaps particularly the ACC and GP, have been under strong evolutionary pressure to function optimally for healthy growth, which has resulted in adaptations leaving them less well‐suited to sustained longevity, leading to age‐related diseases such as OA. This, however, remains to be tested.

## 6 TIDEMARK

Continuous collagen type II fibres abruptly transition from non‐calcified HAC to ACC at the mineralisation front, which is most clearly distinguishable in 3D by quantitative backscattered electron scanning electron microscopy (qBSE‐SEM), μCT or other forms of high‐resolution CT (e.g. synchrotron). This transition can be clearly seen in 2D in haematoxylin and eosin or toluidine blue‐stained histological sections as a basophilic ‘tidemark’ line, which has its own structural and biochemical properties dissimilar from those of the HAC and ACC on either side. Although frequently used synonymously, there are subtle differences between the ‘tidemark’ and the ‘mineralisation front.’ Thus, a joint may have a single mineralisation front where HAC transitions into ACC, but multiple tidemarks visible in the ACC. In this case, deeper tidemarks will have ACC on either side, representing the locations in which a mineralisation front used to exist. Tidemark multiplication occurs as the mineralisation front encroaches into the HAC, causing it to thin unless cartilage expansion at the joint surface is equal to or greater than the encroachment rate. Tidemark multiplication is also referred to as tidemark ‘duplication’ or ‘replication’ in the wider literature. The authors have chosen ‘multiplication’ to clarify that we refer to the process by which multiple tidemarks arise, as opposed to the renewal or doubling of any specific tidemark. The distance between multiplicated tidemarks can vary, but a single given tidemark is ~3–10‐μm thick (Arkill & Winlove, 2008; Lyons et al., 2005) and indiscernible by clinical CT, MRI or X‐ray (Liu et al., 2019). High‐resolution μCT will reveal the gross‐anatomical shape of the mineralisation front, and therefore the most superficial tidemark, as it contains tightly bound calcium among other components (Lyons et al., 2005).

### 6.1 Tidemark mechanics

**FIGURE 3:**
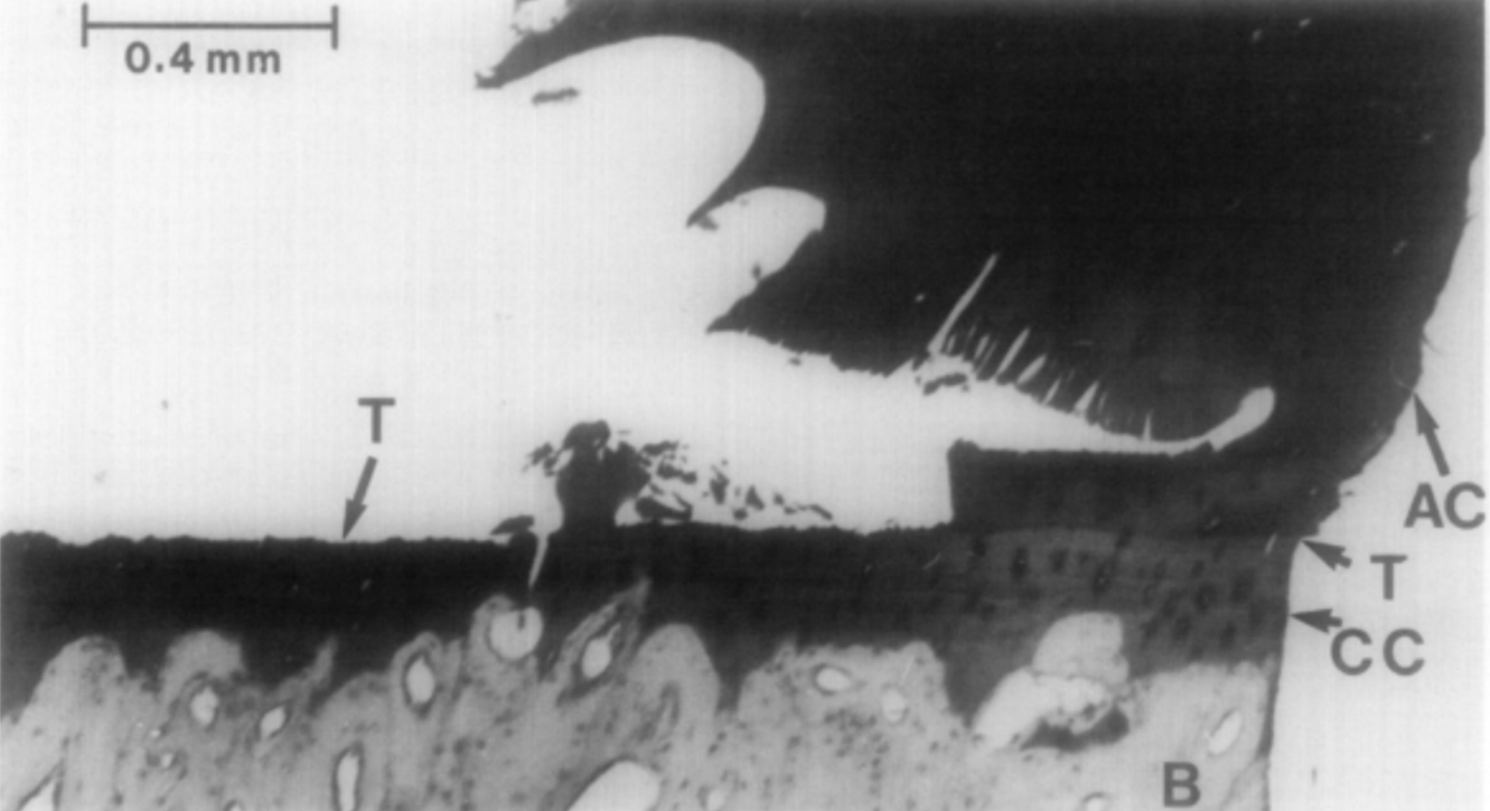
Osteochondral plug from a mature bovine patella. HAC is separated from the ACC at the tidemark (tidemark delamination) under shear applied by mechanical testing (from left to right). Only at the far right‐hand side of the image does the tidemark maintain integrity. AC = articular cartilage (referred to in this review as hyaline articular cartilage), T = tidemark, CC = calcified cartilage (referred to in this review as articular calcified cartilage). Reproduced with kind permission from Broom et al. 1996.

Tidemark function is incompletely defined, and the adhesive and/or mechanical advantages that a sharp mineralisation front interface confers over a smooth gradient alternative is unclear. Indeed, one apparent disadvantage is evidenced in mechanical testing studies where shear forces can readily separate mature HAC from the ACC at this interface due to their calcification disparity (Broom et al., 1996) (Figure 3). In vivo, the peeling of HAC away from ACC at the tidemark is not, however, an often‐realised clinical concern, indicating that the tidemark is successfully protected from such potentially damaging shear forces—indeed, even in osteochondritis dissecans featuring cartilage delamination, the SCB is widely thought to be the primarily affected tissue and is frequently displaced along with neighbouring cartilage, rather than delamination uniformly occurring at either the tidemark or cement line interface, though this can also occur (Andriolo et al., 2020). Lofvall et al. (2018) hypothesised that interface friction may protect joint tissues by enabling shear stresses to instead become purely tensile and compressive during loading and that tidemark undulations reflect and enhance this adaptation. The irregular surface of the tidemark—and the gently wavy entire ACC layer—means that shear force travelling transversely across the osteochondral junction cannot propagate in a quick, straight, ‘shearing’ line, but instead will repeatedly meet hard angles, which redirect or dissipate the forces into a compressive or tensile direction. The osteochondral junction is more able to tolerate these forces without disintegration due to the fact that collagen fibrils are perpendicular to its axis (Redler et al., 1975). Within the extremely slender tidemark itself, collagen fibrils travel in a range of vertical, oblique and irregular directions, forming a ‘net’ which presumably helps to mesh the HAC to the ACC (Chen et al., 2002).

#### 6.1.1 Tidemark advancement

The tidemark is perhaps the most studied ACC region. Tidemark multiplication and advancement into the HAC are well‐established OA hallmarks, linked to SCP thickening (ACC included), and thinning of the HAC. Tidemark advancement can occur over the whole joint, as in healthy, ageing joints in which multiple tidemarks are observed even at OARSI grade 0 (Deng et al., 2016), in models of disuse, or prematurely in specific joint regions with OA. Although tidemark multiplication is not always pathological (Boyde, 2021), tidemark movement into the HAC is accelerated with joint unloading (Nomura et al., 2017; O'Connor, 1997). Additionally, some joints develop highly dense mineralised projections (HDMPs) that project from the ACC into the HAC at a greater distance than typically occurs in tidemark multiplication and can be seen in μCT (Boyde et al., 2014; Laverty et al., 2015). These HDMPs are believed from nanoindentation studies to be stiff and brittle, posing a risk that they may fracture and damage surrounding cartilage (Boyde et al., 2011).

In physiological health, ACC chondrocytes maintain a relatively stable and quiescent hypertrophic state, whereas chondrocytes in OA can stimulate the overproduction of mineralised matrix into the normally uncalcified cartilage, leading it to thin from the osteochondral junction outwards (Oegema Jr. et al., 1997). Different chondrocytes undergoing this behaviour will manifest it diversely, rather than in one single ‘OA signalling/secretory phenotype’—this diversity in synthetic capabilities likely results in observations of heterogeneous matrix quality, ACC susceptibility to local cracking and/or water concentration within the collagenous matrix. The latter of these characteristics is opined to initiate HDMP formation, potentially a process in which a crack initiated in the ACC propagates, breaks through the tidemark and becomes a tear in the HAC. Highly mineralised infill material is then deposited in the ACC crack and so is extruded into the HAC tear with which it is continuous (Boyde, 2021). HDMPs therefore are not surrounded by tidemark (Boyde et al., 2011). The exact changes in the ACC that precipitate HDMP formation are, however, yet to be established.

#### 6.1.2 Tidemark role

Lyons et al. (2005) have proposed the elegant—although currently difficult to test—hypothesis that the ACC tidemark has evolved for the specific purpose of preventing or limiting ACC mineralising front encroachment into the HAC. Following the failure of the initial tidemark in this function, it becomes a ‘non‐functional relic (Lyons et al., 2005),’ and a new tidemark is later formed at the newly developed ACC/HAC interface. This view implies that tidemark multiplication is a later consequence of ACC mineralisation front encroachment, rather than the tidemark being continually a component of the front which itself encroaches (Lyons et al., 2005). Lyons' hypothesis appears consistent with Jiang et al.'s (2008) evidence that HAC chondrocytes inhibit local matrix mineralisation by suppressing ALP activity superficial to the tidemark: the tidemark would provide a convenient boundary at which this HAC chondrocyte influence could be suppressed.

In equines, the region of ACC closest to the tidemark (superficial 50–75 μm) is the most permeable region to solute diffusion (Arkill & Winlove, 2008). A biological, as opposed to mechanical, hypothesis as to the layered nature of the chondro‐osseus junction could be that it facilitates greater control over solute diffusion through the cartilage layers and into the bone and vice versa.

#### 6.1.3 Tidemark composition

Tidemark composition is challenging to evaluate, and researchers doing so have obtained what appear at first glance to be contradictory results. Rhodamine B perfusion and binding research by Arkill and Winlove (2008) appear to confirm that the ACC has an overall anionic fixed charge density, which increases with tidemark proximity, yet Lyons et al. (2005) have described it as lacking the glycosaminoglycans of typical proteoglycans. One possible explanation for this seeming anomaly might be difficulty discriminating between intracellular and extracellular sources of anionic charge (such as DNA and glycosaminoglycans respectively) using perfusion. In light microscopy of stained sections, Lyons et al. (2005) provide evidence of a trilaminate tidemark arrangement, with distal, proximal and central laminae, the latter of which is acknowledged as a potential artefact. Whether these laminae remain intact in the case of tidemark multiplication, or if there are specific consequences to laminar eburnation in late‐stage OA, is unclear. As it is difficult to routinely undertake this level of complex analysis, the most frequently investigated features of the tidemark are its overall multiplication, thickness and roughness, with these investigations performed in research rather than clinical settings. Even Mankin scoring, rare in its consideration of the tidemark at all, allows the tidemark to be either ‘intact’ or ‘destroyed’ with no greater descriptive subtlety available (He et al., 2014).

The possibility that compositional matrix changes at the tidemark may be a component of OA that develops in STR/Ort mice—a popular animal model of human OA—is supported by a greater local abundance of bone sialoprotein‐1 immunolabelling coincident with HAC lesion onset (Mason et al., 2001). Tidemark thickness in a cadaveric study appeared to be highest in ‘moderate OA’ (OARSI score) having increased up until that point and beginning to decline afterwards (Aho et al., 2017).

#### 6.1.4 Tidemark roughness

TMR is frequently assessed in histological studies of animal models with induced OA, and occasionally in CT studies. Despite its ubiquity, methods vary and results are difficult to compare. ‘Roughness’ can be attributed to different tidemark features, including undulation wavelength or surface particularity, depending on the analysis method and image resolution from which roughness of the tidemark surface was calculated.

Although TMR measurements are scientifically illuminating, measurement methods are far from ideal. One method involves drawing a straight line across the tidemark, as seen in histology or CT, and additionally tracing the true line of the tidemark. TMR is then determined either by the difference in length between these lines or the area of space between them (Schultz et al., 2015). It is clear that slice thickness and image resolution will impact these measures, with low resolution better assessing the overall surface undulation and higher resolutions better reflecting smaller tidemark dimples and flakes. Measurements would even be minutely affected by the thickness of the straight line drawn. Differences in large undulations (from low‐resolution CT) versus smaller ‘flaky prominences’ (from SEM; Wang et al., 2009) have been described as ‘low’ and ‘high’ frequency roughnesses respectively (Kauppinen et al., 2019). The difference between the measurements of TMR obtained from high‐ and low‐resolution images could theoretically be calculated as a fractal dimension, although this has not been performed to the authors' knowledge.

Another flaw in the method is that it is not done in 3D, but performed repeatedly on 2D slices, with repetition frequency varying across the literature. Similar to calculations of matrix thickness, TMR is generally calculated from either small osteochondral cores and/or a subsample of histological sections, and a mean value produced in relative units (e.g. between regions of a condyle, or OA/healthy joint samples; Maroudas et al., 1985). A primary limitation to TMR calculation is its failure to account for plateau shape. Additionally, tidemark multiplication is addressed inconsistently between TMR studies and where the tidemark surface is hand‐drawn (Schultz et al., 2015), outcomes will also be influenced by investigators' dexterity and illustrative consistency. To the authors' knowledge, there are no studies in which high‐/low‐frequency tidemark undulations were both assessed to compare their independent contributions to overall TMR. Conclusions drawn as to the cause of any identified changes are therefore limited. Because TMR outcomes tend to be averaged across joint regions, they are rarely integrated with the highly localised anatomical OA‐related changes, such as trabecular changes, GP bridging or chondrocyte clustering.

Despite its limitations, TMR measurement is nonetheless valuable: evidence that it decreases post‐traumatic OA but increases age‐related OA is one of the more subtle indications of the potential value of using ACC as a disease endotype biomarker (Schultz et al., 2015). TMR analysis has additionally confirmed that tidemark undulation is not fixed but responds to mechanical stimuli (Oegema Jr. et al., 1997). Schultz et al. (2015) have indicated that the medial tibial plateau has the least rough tidemark region, raising the exciting possibility of the tidemark having condylar anatomy that reflects regional OA predisposition to onset or progression. Greater consistency of methodology and ease of application in TMR measurement may aid research in this promising direction and elucidate the cause‐and‐effect relationship underlying these observations.

**FIGURE 4:**
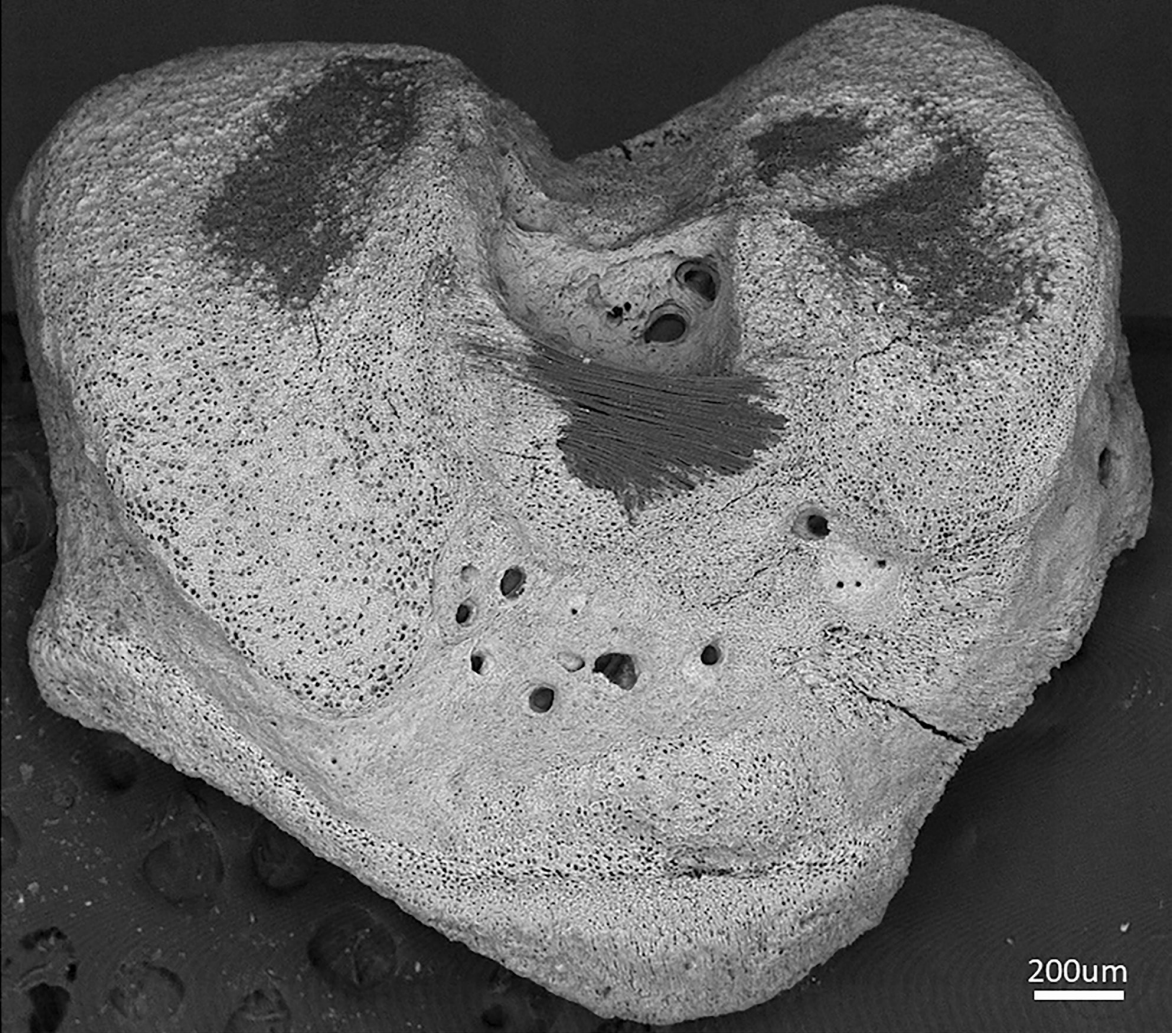
A 10‐week‐old C57/BL6 mouse proximal tibial surface viewed in SEM. HAC has been removed using TergazymeTM (Alconox Inc.: an alkaline bacterial pronase detergent), leaving ACC intact (Boyde 2012). Numerous ‘pits’ can be observed on the superficial ACC surface, consistent with approximate chondrocyte size, indicating that a significant chondrocyte population may exist half‐in and half‐out of the ACC and HAC matrices. These pits were apparent in normal joints at all ages examined (8–40 weeks). Note also the larger fenestrations traversing the epiphyseal bone surface to contact the bone marrow cavity, both in and anterior to the intercondylar space. Reproduced with kind permission from Professor Alan Boyde (unpublished collaboration).

Although rarely observed in histology, removing HAC with proteases reveals a pitted ACC surface in which it appears that chondrocytes are part‐exposed (Oegema Jr. et al., 1997) and part‐enclosed in the matrix (‘half‐in, half‐out’ chondrocytes) (Figure 4). This is in contrast to classical depictions of chondrocytes as generally belonging discretely to one or another specific ‘zone’ of the cartilage. This is an interesting observation that would ideally be better explored with a TMR measurement in 3D, in which a specific ‘frequency’ of undulation could be calculated with the overall joint shape somehow controlled for and analysed independently. Methods to allow for the objective measurement of this particular feature of the ACC tidemark are, however, currently lacking.

**FIGURE 5:**
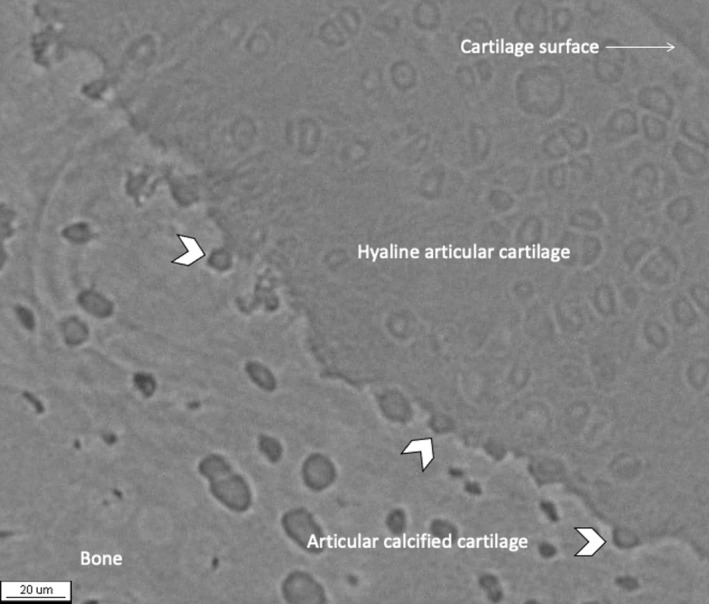
Synchrotron phase‐contrast X‐ray image of a non‐stained, non‐fixed ACC/HAC junction, in which both mineralised and unmineralised tissues are clearly visible. ‘Half‐in, half‐out’ chondrocytes are highlighted by white chevrons. Sample originates from a 40‐week‐old male STR/Ort mouse, the Royal Veterinary College. Half‐in, half‐out chondrocytes were also observed in healthy and younger mice (authors’ own unpublished data).

Structures that appear to be half‐in, half‐out chondrocytes can also be clearly seen using high‐resolution synchrotron phase‐contrast X‐ray imaging (Figure 5, authors' own work, previously unpublished). Although the mineralisation front is clearly visible in these images, the tidemark cannot be clearly discerned, nor could the presence or absence of multiple tidemarks. The observation of these cells therefore raises questions about 3D tidemark anatomy. Is the tidemark consistently deep to the cells (such that they could be considered HAC chondrocytes), consistently superficial to them (such that they could be considered ACC chondrocytes), or neither? Does the tidemark perhaps have sporadic full‐thickness perforations throughout its surface, within which the chondrocytes can nestle? If so, does this have consequences for biochemical permeability or for joint interface integrity under shear force? Are the chondrocytes themselves polarised, with differing basal/apical phenotypes in terms of molecular secretions and signalling? Is it at all possible to extract these cells specifically from a joint and observe their behaviour in cell culture? Lack of understanding about this population of chondrocytes is one of the many ways in which the regulation of the HAC‐to‐ACC transition is poorly understood.

## 7 CEMENT LINE

**FIGURE 6:**
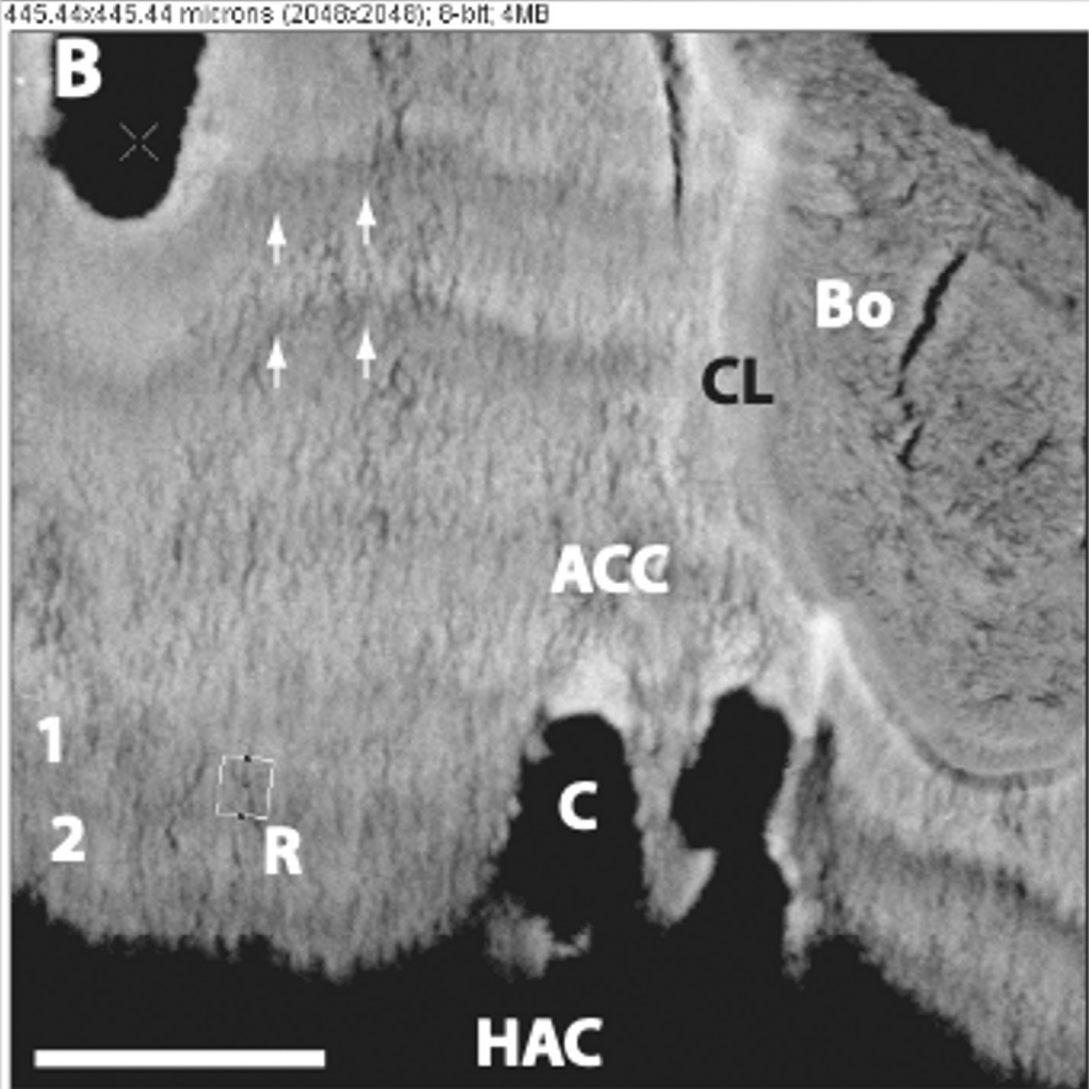
qBSE image clearly showing the cement line (CL). Scale bar = 30 μm. Bo, bone; ACC, articular calcified cartilage; C, chondrocyte; HAC, hyaline articular cartilage; R, a region of interest; 1 and 2, locations of calcein labels not visible in greyscale. Arrows demonstrate multiple tidemarks. Reproduced with kind permission from Doube (2008).

Like the mineralising front but unlike the tidemark, the cement line is defined more by being an interface than a discrete anatomical structure in its own right, although focused‐ion beam‐scanning electron microscopy research by Tang et al. (2022) suggests that it may have unique properties at the nanoscale level. The cement line interface between ACC and bone is characterised by changes in collagen fibre type (type II in cartilage vs type I in bone) and the orientation of both mineral particles (Zizak et al., 2003) and collagen fibres (perpendicular to the joint surface in ACC vs parallel to it in bone; Arkill & Winlove, 2008): it is therefore obvious when viewed in backscattered electron SEM (Figure 6) or histologically using a Masson trichrome stain (Kauppinen et al., 2019). CT offers a non‐destructive alternative (Bailey & Mansell, 1997) when imaging modality and settings are sensitive enough to resolve small shifts in mineral concentration (Rytky et al., 2021). When this is not the case, as the visible mineralisation difference between ACC and SCB is very small, even μCT studies tend to ignore the cement line rather than perform complicated and time‐consuming segmentations. Grouping ACC and SCB together as one SCP for analyses is an unfortunate predicament for OA research, as it would be preferential if bone and cartilage were both studied separately and thoroughly. Rytky et al. (2021) have contributed to addressing this, in their automated deep‐learning pipeline that allows segmentation of ACC in small image volumes with isotropic pixel sizes at least up to 3.2 μm. This allows small volumes of ACC thickness and chondrocyte characteristics to be objectively measured and compared and can be anticipated to enrich future SCP‐focused μCT studies. When ACC and SCB are grouped together, researchers are likely to conclude that bone is more porous than is in fact the case (due to hypertrophic ACC lacunae), or that depth‐dependent mineralisation changes occur in bone, when in fact these may be a false signal derived from ACC changes. Similar errors could arise from simple volumetric measurements—for example sclerosis of the SCB may be allied to ACC erosion in a given pathology or animal model—yet it could easily be concluded that there has been no SCP change, or the magnitude of the change could be underestimated, from analysis of CT data which lumps the two zones together. Likewise, analysis of the ‘whole’ articular cartilage is diminished in richness by loss of its deepest zone from all consideration, resulting in the current situation in which HAC is far better characterised than its mineralised neighbour, and separate, more challenging studies of isolated ACC need to now redress the imbalance. Anatomical assertions about ‘chondrocytes’ as a whole, including those made herein on the basis of HAC observations, may yet transpire to be depth‐dependent.

Although also undulating, the cement line is most frequently described as a ‘less rough’ or smoother (‘comb‐anchor’ shape) interface than the tidemark (Schultz et al., 2015; Wang et al., 2009). When Deng et al. (2016) measured cement line roughness, they found that it correlated with the thickness of both SCB and HAC, in human OA total knee replacement samples from grade 0 to grade 3.

### 7.1 Cement line function

As well as maintaining osteochondral integrity, the cement line (sometimes even the entire ACC itself) is regarded as preventing over‐diffusion of nutrients from the marrow cavity into the HAC; ACC is fivefold less permeable to some solutes than the HAC (Arkill & Winlove, 2008). In return, the ACC also limits available nutrition from synovial fluid that is available to bone and its own deeper cells (Oegema Jr. et al., 1997), which may be a protective feature in OA and rheumatoid joint diseases in which synovial fluid contains an excess of cytokines and growth factors. ACC also regulates the perfusion of smaller molecules. In this way, the ACC may serve a metabolic role in bone–cartilage crosstalk, in addition to its adhesive and biomechanical roles. Interestingly, in the ‘whole joint’ context, ACC permeability is associated closely with its mechanical environment: static loading has been shown to reduce ACC permeability to the chemical diffusion of small anions (Arkill & Winlove, 2008).

The view of ACC existing as a carefully regulated nutrient barrier is challenged, however, by the existence of infrequent fenestrations through the ACC which allow HAC to directly communicate with bone marrow cavities in some regions (via a boundary consisting only of intact tidemark)(Lyons et al., 2006). In OA samples, some of these perforations are undoubtedly a reflection of pathological cartilage vascularisation, allowing blood vessels to pass through into the HAC, including through breaches of the tidemark (Suri et al., 2007). Some of these can, however, also be observed in healthy human cadaveric specimens (Duncan et al., 1987). Boyde (2021) describes the origin of at least some fenestrations as due to osteoclastic ‘cutting cones’ in the SCB which, on occasion, penetrate too far and inappositely puncture the ACC full‐thickness, resulting in the observed hole, and a region in which HAC is disconnected from bone. This has been observed to occasionally occur even in healthy joints (Clark, 1990), although its occurrence is more widespread in OA (Botter et al., 2011; Hwang et al., 2008). The resulting fenestration is less readily filled‐in by remodelling activity than bone, and there is currently no evidence that ACC chondrocytes possess repair‐associated mechanisms with which to signal local damage, as are reported to exist in osteocytes (Schaffler & Kennedy, 2012) and the chondrogenic progenitor cells of the superficial HAC (Ding et al., 2021), nor that if they did, they would be capable of migrating to a repair site.

Not to be confused with small, articular surface perforations, long bones also contain larger, macroscopically obvious fenestrations located outside the articular region—using the proximal tibial epiphysis as an example, they can be seen in the bone of the intercondylar region and anterior to the condyles (Matsuo et al., 2019), and they do not appear to traverse the ACC, although some do have peripheral edges in contact with it (Figure 4). These have been referred to as Lijianmin–Chengkun complexes (Shao et al., 2021), and the combined work of Matsuo et al. (2019) and Shao et al. (2021) show them to be neurovascular foramina.

Pathological vascular ingrowth, with linked osteoclastic resorption, into the HAC in OA initially occurs through the cement line, a region directly deep to which there are normally many blood vessels nourishing the SCB (Clark, 1990). This invasion into the HAC may also be facilitated by chondroclasts, potentially tied to the neural invasions that result in OA pain, by creating the required passageways through tissues (Suri et al., 2007). Angiogenesis itself may be a response to inappropriate chondrocyte signalling, as altered morphology in early OA markedly alters their signalling output (Hall, 2019). When chondrocytes have apoptosed or chondroptosed, the cellular debris is removed by blood vessels (Xie et al., 2020) or macrophages (Pazzaglia et al., 2020). The relationship between levels of both chondrocyte hypertrophy and angiogenesis is clearly intimate and complex.

## 8 CONCLUSIONS

Viewing ACC as a mere remnant of endochondral ossification fails to address its ubiquity, nor to explain the active metabolic changes it exhibits in response to environmental challenges. ACC may exist for many reasons, including the maintenance of its interface integrities. ACC load transmission appears likely to play a role in regulating SCB remodelling. Research is needed to identify whether this is actively beneficial, or merely a consequence of the layer's location. This is not to denote ACC as an ‘ideal’ tissue, as it may function better in growth stages than in sustained longevity, and in this way contribute directly to the OA explosion in our ageing population. Any given OA joint is likely to contain a range of pathologies (e.g. osteophytes and eburnation are often spatially separated from one another; Hayeri et al., 2010). While ACC is a promising biomarker, it is not clear whether it can serve as an OA biomarker, or of specific features of independent processes affecting the bone–cartilage unit. Ken Brandt (providing the opposition view in Guermazi et al.’s, 2013 debate paper) points out that with the recent improvement in our understanding of OA as both a whole organ disease, and an individualistic one, it is possible that any tissue may contain the initiating factor in any given patient and yet affect mechanics in such a way that OA is consistently the end process (i.e. that any tissue could be the first to fail or to initiate total joint failure). In the same vein, however, any given tissue, including ACC, may be a necessary ‘midpoint’ for OA development to surpass a given threshold for progression, and thus comprise a meaningful pharmaceutical target. Better, more thorough and standardised evaluation of ACC matrix and cell changes may pinpoint the similarities and differences between animal OA models that exploit different species, surgeries, age of onset, loading regimens, etc—to confer meaningful comparisons and enhanced understanding of how to translate OA changes into ‘one medicine’ clinical/veterinary practice. It is also apparent that consistency in the linguistic delineation of ACC and its components will enable superior meta‐analysis of ACC‐related literature. Likewise, greater consistency of research methods in ensuring that ACC is separated from its HAC and SCB neighbours during analyses will enrich scientific and anatomical understanding of all three joint compartments in health and disease.

## AUTHOR CONTRIBUTIONS

Lucinda AE Evans – first draft and multiple subsequent rewrites. Andrew A Pitsillides – contextual and structural feedback, substantive rewrites of multiple sub‐sections.

## FUNDING INFORMATION

The authors did not receive support from any organisation for the submitted work, but the ability to complete it depended in part upon the following grant: Anatomical Society ‘How does joint anatomy resist cracking under pressure’, PI Andrew Pitsillides, Royal Veterinary College.

## CONFLICT OF INTEREST

The authors have no conflict of interest to declare that are relevant to the content of this article.

## CONSENT FOR PUBLICATION

The authors’ consent to the publication of the submitted content for which they hold copyright in all instances.

## Data Availability

N/A

## Endnote

1 The physiologic death of hypertrophic chondrocytes has been conflated with ‘apoptosis’ in the literature, likely because both processes constitute non‐necrotic, controlled cell death. Another reason may be an inadequacy of standard laboratory staining and other methods to clearly discriminate between these two types of cell death—for example, DNA cleavage occurs in both, therefore, will stain positive in a TUNEL assay (Roach & Clarke, 2000; Roach et al., 2004). Classical apoptosis is preceded by cell shrinkage due to the loss of ions and thus water with reference to K^+^ and Cl^−^ ion transporters (Hall, 2019), however, chondroptosis is the inverse, being a process of death that involves increased protein production (Roach et al., 2004) and hypertrophic growth, or NKCC cotransporter stimulation leading to cell swelling (Bush et al., 2010). See Roach et al. (2004) and Hall (2019) for more details on the topic and information on the two types of death.
